# Filamentation of Metabolic Enzymes in *Saccharomyces cerevisiae*

**DOI:** 10.1016/j.jgg.2016.03.008

**Published:** 2016-06-20

**Authors:** Qing-Ji Shen, Hakimi Kassim, Yong Huang, Hui Li, Jing Zhang, Guang Li, Peng-Ye Wang, Jun Yan, Fangfu Ye, Ji-Long Liu

**Affiliations:** a Medical Research Council Functional Genomics Unit, Department of Physiology, Anatomy and Genetics, University of Oxford, Oxford OX1 3PT, UK; b Key Laboratory of Entomology and Pest Control Engineering, College of Plant Protection, Southwest University, Chongqing 400715, China; c Key Laboratory of Soft Matter Physics, Beijing National Laboratory for Condensed Matter Physics, Institute of Physics, Chinese Academy of Sciences, Beijing 100190, China; d CAS-MPG Partner Institute for Computational Biology, Shanghai Institutes of Biological Sciences, Chinese Academy of Sciences, Shanghai 200031, China

**Keywords:** CTP synthase, Metabolic enzyme, Cytoophidium, Glycolysis, Glutamine, Intracellular compartmentation, *Saccharomyces cerevisiae*

## Abstract

Compartmentation *via* filamentation has recently emerged as a novel mechanism for metabolic regulation. In order to identify filament-forming metabolic enzymes systematically, we performed a genome-wide screening of all strains available from an open reading frame-GFP collection in *Saccharomyces cerevisiae*. We discovered nine novel filament-forming proteins and also confirmed those identified previously. From the 4159 strains, we found 23 proteins, mostly metabolic enzymes, which are capable of forming filaments *in vivo*. *In silico* protein-protein interaction analysis suggests that these filament-forming proteins can be clustered into several groups, including translational initiation machinery and glucose and nitrogen metabolic pathways. Using glutamine-utilising enzymes as examples, we found that the culture conditions affect the occurrence and length of the metabolic filaments. Furthermore, we found that two CTP synthases (Ura7p and Ura8p) and two asparagine synthetases (Asn1p and Asn2p) form filaments both in the cytoplasm and in the nucleus. Live imaging analyses suggest that metabolic filaments undergo sub-diffusion. Taken together, our genome-wide screening identifies additional filament-forming proteins in *S. cerevisiae* and suggests that filamentation of metabolic enzymes is more general than currently appreciated.

## Introduction

Compartmentation of biological processes is a fundamental feature of the cell. One such way is to form membrane-bound organelles, which have been extensively appreciated in the past. Moreover, macromolecules can be compartmentalized through the formation of large-scale aggregates without membranes (Gall, 2000, Brangwynne et al., 2009, O'Connell et al., 2012, Hyman et al., 2014). Emerging studies have identified many membraneless organelles including cytoplasmic processing bodies (P bodies) (Sheth and Parker, 2006), histone locus bodies (HLBs) (Liu et al., 2006a, Liu et al., 2006b), uridine-rich small nuclear ribonucleoprotein bodies (U bodies) (Liu and Gall, 2007) and purinosomes (An et al., 2008).

In 2010, three groups independently described that the metabolic enzyme CTP synthase (CTPS) can form filamentous structures in bacteria, yeast and fruit flies (Ingerson-Mahar et al., 2010, Liu, 2010, Noree et al., 2010). These filaments have been termed as “cytoophidia” (meaning cellular snakes in Greek), “CTPS filaments”, or “cytoplasmic rods and rings”. Subsequent studies have shown that CTPS can form filaments in human cells as well (Carcamo et al., 2011, Chen et al., 2011). Thus cytoophidia represent a novel type of evolutionarily conserved organelles. Recent studies suggest that polymerisation of CTPS or other metabolic enzymes into filamentous structures serves to regulate enzymatic activity (Aughey et al., 2014a, Aughey et al., 2014b, Barry et al., 2014, Noree et al., 2014, Petrovska et al., 2014, Strochlic et al., 2014, Chang et al., 2015). Studies in *Drosophila* indicate that the CTPS cytoophidia are involved in brain development and oogenesis (Chen et al., 2011, Azzam and Liu, 2013, Strochlic et al., 2014, Tastan and Liu, 2015a, Tastan and Liu, 2015b, Wang et al., 2015). The biology of cytoophidia emerges as a new frontier in the field of pyrimidine metabolism (Aughey et al., 2014a, Aughey et al., 2014b, Garavito et al., 2015, Liu, 2015, Tastan and Liu, 2015a, Wang et al., 2015).

Nine filament-forming proteins including CTPS were identified *via* a screening of 1632 GFP-tagged yeast strains (Noree et al., 2010), which comprise about 40% of the budding yeast GFP-tagged open reading frame (ORF) collection (Huh et al., 2003). To identify additional novel filament-forming proteins in budding yeast, we screened the whole collection of 4159 GFP-tagged ORFs, which represents 75% the *Saccharomyces cerevisiae* proteome. From this, we identified 23 proteins (including nine novel proteins) that can form filaments *in vivo* in diauxic and stationary phases. We found that these filament-forming proteins can be clustered into several groups, including translational initiation machinery and glucose and nitrogen metabolic pathways. Further analysis of the five glutamine-utilising enzymes demonstrated that the occurrence and length of the metabolic filaments are sensitive to growth conditions. In addition, we observed that four glutamine-utilising enzymes can form filaments both in the cytoplasm and in the nucleus. Live imaging analysis of six types of filament suggests that they undergo sub-diffusion. The identification of additional filament-forming proteins from our genome-wide screening provides an opportunity to study compartmentation *via* filamentation systematically.

## Results

### Filament-forming proteins in budding yeast

**Fig. 1:**
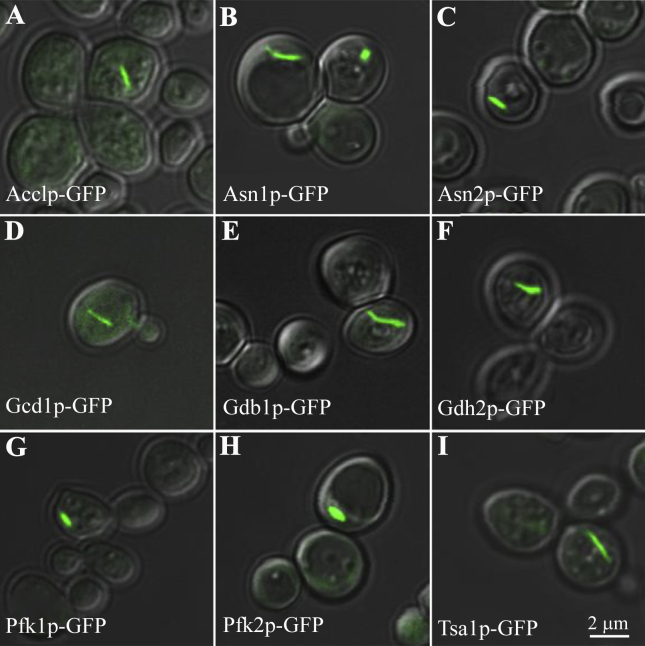
Identification of filament-forming proteins in *S. cerevisiae*. A genome-wide screening of 4159 GFP-tagged ORF collection in budding yeast identifies nine novel filament-forming proteins. **A:** Acetyl-CoA carboxylase (Acc1p). **B:** Asparagine synthetase 1 (Asn1p). **C:** Asparagine synthetase 2 (Asn2p). **D:** Gamma subunit of the translation initiation factor eIF2B (Gcd1p). **E:** Glycogen debranching enzyme (Gdb1p). **F:** Glutamate dehydrogenase (Gdh2p). **G:** Phosphofructokinase (Pfk1p). **H:** Phosphofructokinase (Pfk2p). **I:** Thioredoxin peroxidase (Tsa1p). Scale bar, 2 μm. See also Fig. S1 and Table 1.

Our screening has confirmed that all nine proteins identified in Noree's study (Noree et al., 2010) (i.e., Glt1p, Psa1p, Ura7p, Ura8p, Gcd2p, Gcd6p, Gcd7p, Gcn3p and Sui2p) (Fig. S1A) and all four septin proteins (i.e., Cdc10p, Cdc11p, Cdc12p and Shs1p) (Fig. S1B) available in the budding yeast GFP-tagged ORF collection can form filaments. Short filaments and foci assembled by Gln1p (glutamine synthase) could be detected (Fig. S1C), but there were no long filaments, even after starvation treatment, in the current study, with a potential interference from the GFP tag as reported previously (Petrovska et al., 2014). In addition, nine more proteins can form large-scale filaments detectable under light microscopy (Fig. 1 and Table 1), namely Acc1p (acetyl-CoA carboxylase), Asn1p/Asn2p (asparagine synthetase), Gcd1p (eIF2B-γ), Gdb1p (glycogen debranching enzyme), Gdh2p (glutamate dehydrogenase), Pfk1p/Pfk2p (phosphofructokinase) and Tsa1p (thioredoxin peroxidase). To simplify the terminology, we refer to these metabolic enzyme-containing serpent-shaped structures as cytoophidia.

Acetyl-CoA carboxylase catalyses the carboxylation of acetyl-CoA to produce malonyl-CoA, which provides the malonyl-CoA substrate for fatty acid biosynthesis. In mammals, acetyl-CoA carboxylase can be polymerised into tiny filaments detectable under electron microscopy (Kleinschmidt et al., 1969, Meredith and Lane, 1978). Polymerisation of acetyl-CoA carboxylase upregulates the enzymatic activity. A recent study reported that Acc1p has diffused distribution under normal growth conditions, while prolonged starvation can drive Acc1p to form rod-like structures in budding yeast (Suresh et al., 2015). Our screening revealed that Acc1p is capable of forming large-scale filaments under normal growth conditions.

Using aspartate and glutamine as the substrates, asparagine synthetase catalyses an ATP-dependent reaction to produce asparagine. In budding yeast, there are two genes, *ASN1* and *ASN2*, which encode asparagine synthetase (Dang et al., 1996). We found that Asn1p and Asn2p have very similar distributions and both can form filaments *in vivo*. Studies in *Escherichia coli* and beef pancreas suggest that asparagine synthetase functions as a dimeric enzyme (Gantt and Arfin, 1981, Larsen et al., 2000). Lack of asparagine synthetase may cause cell apoptosis (Zhang et al., 2014a). Due to its important role in amino acid synthesis, asparagine synthetase is a common target in the treatment of acute lymphoblastic leukaemia as well as prostate cancer and other kinds of cancer (Aslanian et al., 2001, Sircar et al., 2012, Panosyan et al., 2014).

Noree et al. (2010) have shown that five subunits of the eIF2 and eIF2B complexes, Gcd2p (eIF2B-δ), Gcd6p (eIF2B-*ɛ*), Gcd7p (eIF2B-β), Gcn3p (eIF2B-α), and Sui2p (eIF2-α), are present in the same filament. In this study, we identified an additional subunit of the eIF2/2B complex, Gcd1p (eIF2B-γ), which can form a similar filamentous structure. Although GFP-tagged Gcd1p (eIF2B-γ) was previously reported as foci in budding yeast (Campbell et al., 2005), the images in that paper showed filament-like structures which are in line with the results of the current study.

Glycogen is a multibranched polysaccharide of glucose residues, which acts for the energy storage in animals and yeast. The accumulation of glycogen responds to nutrient restriction, and to heat, osmotic and saline stress. Glycogen is rapidly mobilised by two enzymes, glycogen phosphorylase and glycogen debranching enzyme when non-proliferating yeast cells resume growth. The glycogen debranching enzyme Gdb1p was identified by Noree et al. (2010) as a foci-forming protein that is incapable of forming filaments. However, our screening indicated that Gdb1p is able to form filaments under normal growth conditions. Gdb1p contains two enzymatic activities, α-1,4-glucanotransferase and α-1,6-glucosidase. It is interesting to point out that filamentation of β-glucosidase was first observed in electron micrographs of oat plastids in 1965 (Gunning, 1965). More recently, biochemical analyses have suggested that the filament is the active form of the β-glucosidase (Kim et al., 2005).

In budding yeast, *GDH1* and *GDH3* encode two NADP^+^-dependent glutamate dehydrogenases (NADP-GDHs) which catalyse the synthesis of glutamate from ammonium and α-ketoglutarate. *GDH2* encodes NAD^+^-dependent glutamate dehydrogenase (NAD-GDH), which degrades glutamate and produces ammonium and α-ketoglutarate (DeLuna et al., 2001). Our screening identified that Gdh2p, but not Gdh1p or Gdh3p, can form filaments *in vivo*.

Phosphofructokinase 1 catalyses the phosphorylation of fructose 6-phosphate to fructose 1,6-bisphosphate. This reaction is the principal regulatory step in glycolysis. The yeast ortholog of human phosphofructokinase-1 consists of heterooctamers α_4_β_4_. Pfk1p (α subunit) and Pfk2p (β subunit) are homologous to each other. Under electron microscopy, cross-linked rabbit muscle Pfk1p has been observed to form dimeric, tetrameric, octameric and filamentous forms *in vitro* (Telford et al., 1975). Immunofluorescence images have shown the filamentous arrangement of Pfk1p in budding yeast (Schwock et al., 2004). In this study, we found that both Pfk1p and Pfk2p could form filaments in budding yeast *in vivo*. It appears that the filament-forming property of phosphofructokinase is evolutionarily conserved and ancient.

Oxidative stress results from oxygen metabolism and occurs in the cells of all aerobic organisms. Oxygen-utilising cells have evolved defence mechanisms called the oxidative stress response (OSR), to protect against the damage caused by oxidative stress. Thioredoxin peroxidase, Tsa1p, plays an important role in OSR. Our screening revealed that Tsa1p can form filaments *in vivo*.

### Clusters of filament-forming proteins

**Fig. 2:**
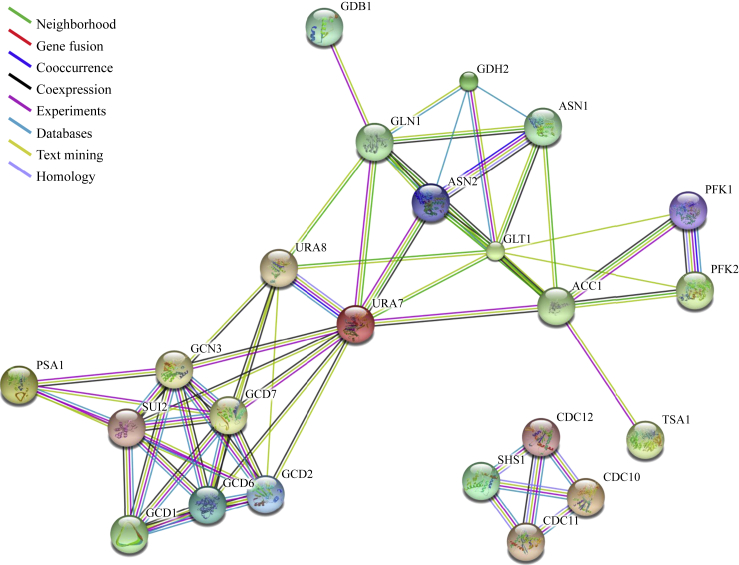
Protein-protein analysis of filament-forming proteins in budding yeast. The high-resolution evidence-view of networks was obtained with STRING under medium confidence 0.400. Protein nodes indicate the availability of 3D protein structure information and coloured lines between the proteins indicate the various types of interaction evidence. See also Table 2.

Network analysis of proteins is useful to know how these proteins interplay with other key proteins and pathways. To assess the relationships among these 23 filament-forming proteins (Table 1), the protein-protein interaction analysis was performed by utilising the STRING database version 10 (Szklarczyk et al., 2015). Coloured lines between the proteins represent the different types of interaction evidence, and neighbourhood, cooccurrence, experiments, text mining, coexpression, databases and homology were utilised in the analysis (Fig. 2). The results illustrate the STRING gene set enrichments for those filament-forming proteins against related KEGG pathways with significance rank *P* < 0.05 after correction by false discovery rate (FDR). As expected, the septin complex proteins Cdc10p, Cdc11p, Cdc12p and Shs1p are clustered together. Enrichments on the eIF2/2B complex (Gcd1p, Gcd6p, Gcd2p, Gcd7p, Gcn3p and Sui2p) indicate the strong association with the pathway of RNA transport (*P* = 1.11 × 10^−5^). Five proteins (Asn1p, Asn2p, Gdh2p, Gln1p and Glt1p) were found to be associated with alanine, aspartate and glutamate metabolism (*P* = 4.34 × 10^−6^). Amongst these five proteins, Gdh2p, Gln1 and Glt1 were closely related to nitrogen metabolism (*P* = 7.54 × 10^−5^). Apart from the septin proteins and eIF2/2B complex, 12 of the other 13 proteins were enriched in metabolic pathways (*P* = 5.55 × 10^−5^), with Pfk1p, Pfk2p and Psa1p associated with fructose and mannose metabolism (*p* = 7.33 × 10^−3^) (Table 2).

### Heterogeneities of cytoophidia in *S. cerevisiae*

**Fig. 3:**
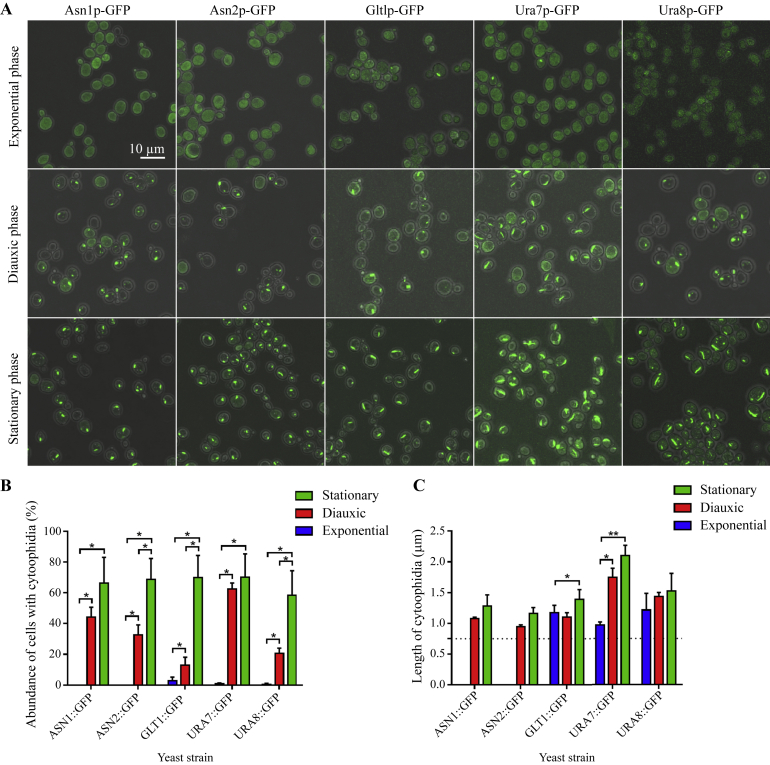
The assemblies of glutamine-dependent metabolic enzymes into cytoophidia at different growth phases. **A:** Photomicrographs of yeast cells displaying gradual changes in the abundance and length of cytoophidia in exponential phase, diauxic phase and stationary phase. Scale bar, 10 μm. **B:** Averaged abundance of cytoophidia formed by each enzyme. **C:** Averaged length of cytoophidia with the cutoff value of 0.75 μm. Data are represented as mean ± SD. *, *P* ≤ 0.05 and **, *P* ≤ 0.01.

In budding yeast, there are three distinct growth phases that are coupled with different metabolic signatures. However, the correlation between distinct growth phases and the regulation of cytoophidia in yeast cells remains unclear. To investigate the relationship between these two parameters, we cumulatively selected five cytoophidium-forming enzymes that are involved in amino acid (Asn1p, Asn2p and Glt1p) and pyrimidine (Ura7p and Ura8p) metabolisms, respectively. As expected, the formation of cytoophidia was gradually elevated from exponential to stationary phases (Fig. 3A).

Quantitative analyses of cytoophidia abundance displayed an increasing pattern of propensity of all five metabolic enzymes to form cytoophidia from exponential to diauxic and stationary phases (Fig. 3B). In exponential phase cultures, Asn1p and Asn2p did not coalesce into cytoophidia, whereas there was only a very low abundance of cytoophidia formed by Glt1p, Ura7p and Ura8p, respectively. These five enzymes form obvious filamentous structures in the diauxic phase. The abundance of cytoophidia increases significantly from the exponential phase to the diauxic phase and from the diauxic phase to the stationary phase. In general, all five enzymes displayed highest abundance of cytoophidia in stationary phase as compared to other growth phases.

The length of cytoophidia formed by Glt1p or Ura7p was evidently increased as the cells rested in the stationary phases, while Asn1p, Asn2p and Ura8p form cytoophidia with comparable length between the diauxic phase and the stationary phase (Fig. 3C). Collectively, these data indicated that enzymes involved in amino acids (Asn1p, Asn2p and Glt1p) and pyrimidine (Ura7p and Ura8p) biosynthetic pathways orchestrated heterogeneity in terms of the regulations of cytoophidia in different growth phases.

### Filamentation in the nucleus

**Fig. 4:**
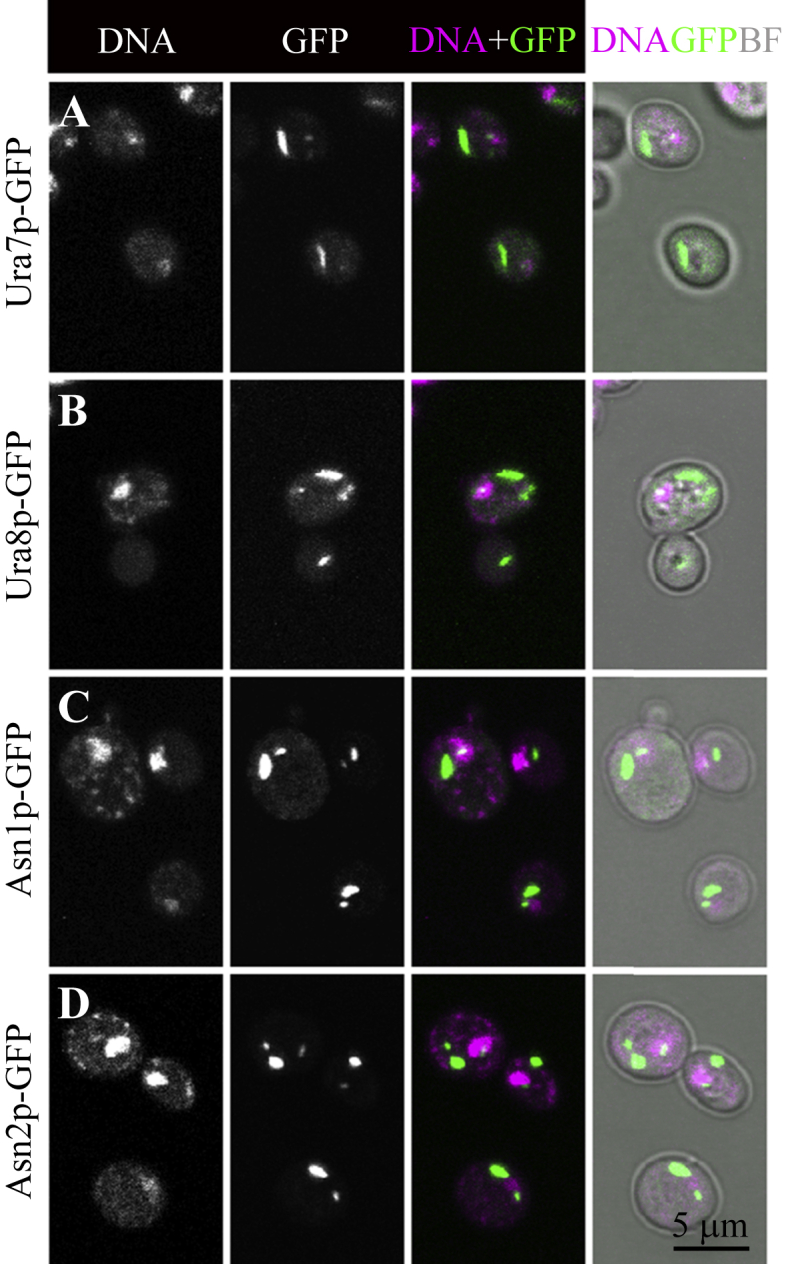
Filaments formed in the cytoplasm and nucleus. Budding yeast cells expressing GFP-tagged ORFs were observed under confocal microscopy. Both isoforms of CTPS, Ura7p (**A**) and Ura8p (**B**), can form cytoophidia in the cytoplasm and in the nucleus, consistent with our previous findings in *S. pombe* (Zhang et al., 2014b) and mammalian cells (Gou et al., 2014). Both isoforms of asparagine synthetase, Asn1p (**C**) and Asn2p (**D**), can also form cytoophidia in the cytoplasm and in the nucleus. Note that cytoplasmic cytoophidia are longer and thicker than nuclear cytoophidia. Scale bar, 5 μm.

We previously found that CTPS can form both cytoplasmic cytoophidia (C-cytoophidia) and nuclear cytoophidia (N-cytoophidia) in mammalian cells (Gou et al., 2014). In addition, we observed that CTPS can form C- and N-cytoophidia in the fission yeast *Schizosaccharomyces pombe* (Zhang et al., 2014b). Noree et al. (2010) observed that the two CTPS proteins, Ura7p and Ura8p, can form filaments in budding yeast. However, whether Ura7p and Ura8p form structures in the nucleus remained unexplored. To address this issue, we closely looked at Ura7p-GFP and Ura8p-GFP cells counterstained with the DNA dye Hoechst 33342. Our examination suggests that both Ura7p-GFP and Ura8p-GFP can form filamentous structures in the nucleus, as well as in the cytoplasm (Fig. 4A and B). Our results regarding the subcellular distribution of CTPS in *S. cerevisiae, S. pombe* and mammalian cells indicate that both C- and N-cytoophidia are highly conserved during evolution. Encouraged by these observations, we carefully checked other filament-forming proteins in budding yeast. We found that both Asn1p and Asn2p can form nuclear filaments as well as cytoplasmic filaments (Fig. 4C and D).

### Dynamics of metabolic filaments

**Fig. 5:**
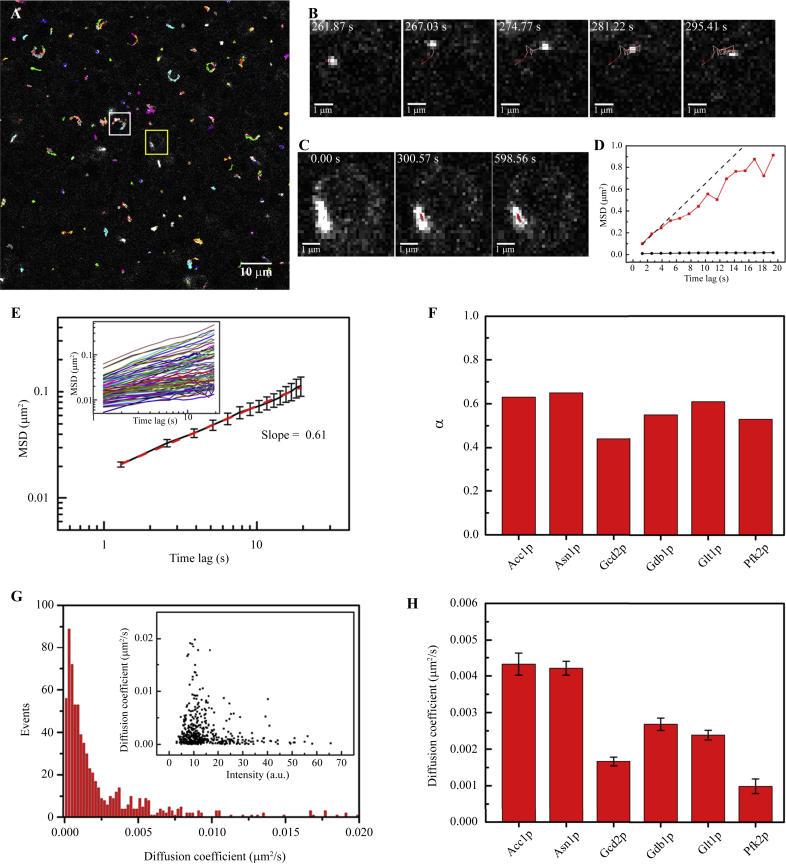
Dynamic analysis of cytoophidia. **A:** Representative trajectories of the Glt1p cytoophidia in budding yeast from 10 min time-lapse movies at room temperature. The paths are marked by colour lines. **B** and **C:** Image series of the Glt1p cytoophidia in the rectangular regions (white box (**B**), yellow box (**C**)) marked in (**A**). **D:** Plots of the mean square displacement (MSD) curves for the trajectories in (**B**) and (**C**). The red solid line (corresponding to the cytoophidium in (**B**)) is lower than that of a free diffusion (indicated by the dash line), showing that the cytoophidium movement in (**B**) is sub-diffusion. The black solid line (corresponding to (**C**)) remains almost unchanged over time lag, showing the cytoophidium in (**C**) is confined. **E:** Averaged MSD of ∼700 Glt1p cytoophidia as a function of time lag has an exponent α about 0.61 (given by the slope of the plot), suggesting the motion type of Glt1p cytoophidia is sub-diffusion. Inset, ∼100 samples of MSD curves. **F:** Comparison of averaged α values for six kinds of cytoophidia: Acc1p (*n* = 122), Asn1p (*n* = 376), Gcd2p (*n* = 289), Gdb1p (*n* = 266), Glt1p (*n* = 700), and Pfk2p (*n* = 64). **G:** Distribution of the diffusion coefficients for the trajectories of Glt1p cytoophidia (*n* = 700). The inset shows the relation between the diffusion coefficients and the fluorescence intensities for Glt1p cytoophidia. Most Glt1p cytoophidia have diffusion coefficient <0.0025 μm^2^/s, whereas only a few ones with relatively low intensities have larger coefficients. **H:** Comparison of diffusion coefficients of six kinds of cytoophidia, with the same sample size as (**F**). See also Data in Brief (Li et al., 2016).

To understand the behaviour of these filaments, we performed live imaging with *S. cerevisiae* cells expressing various filament-forming proteins fused with GFP. As was shown above, only in the diauxic and stationary phases of budding yeasts the cytoophidia were abundant and apt to be studied, therefore, we chose the budding yeasts in diauxic and stationary phases and diluted with PBS to maintain the state of cytoophidia for the following experiments. The live cells were recorded every 1.29 s for 10 min. For example, tracking of the Glt1p filaments showed that these structures are motile and located preferentially at the cell cortex (Fig. 5A). A closer look at these filaments shows that some of them are highly dynamic (Fig. 5B), whereas others are relatively slow (Fig. 5C).

Generally, we selected six types of filament-forming proteins for further in-depth analysis (Fig. 5; Data in Brief see Li et al., 2016). We investigated how the mean square displacement (MSD) of the filaments varies with time lag τ. For a free random thermal diffusion, a linear relationship between the MSD and τ is expected, i.e., $\text{MSD}\propto \tau^{\alpha }$, with *α* = 1; for a thermal diffusion confined in certain areas, the value of *α* is expected to be smaller than 1; if the motion of the filaments is driven by motor proteins, the filaments would exhibit active transport, yielding an *α* of value larger than 1. We started by analysing the MSD curves of the two filaments shown in Fig. 5B and C and found that they were both downward compared with the dashed line (corresponding to pure random diffusion), suggesting the filaments underwent sub-diffusion, i.e., random diffusion within confined areas (Fig. 5D). The averaged MSD of the ∼700 Glt1p filaments as a function of time lag was shown in Fig. 5E, whose exponent *α* is 0.61 (given by the slope of the plot), supporting the theory that the motion type of the Glt1p filaments is sub-diffusion. For all six types of filament-forming proteins analysed, we found that their *α* values were comparable (between 0.38 and 0.65) (Fig. 5F), implying that the dynamics of these filaments formed by different proteins all exhibit sub-diffusion behaviour, and are not driven by motor proteins. This conclusion support a previous study on protein aggregates in budding yeast (Zhou et al., 2011). We are, however, aware that this conclusion is based on our time resolution (1.29 s/frame) and that burst active transports (e.g., based on the actin cytoskeleton) shorter than this time resolution cannot be ruled out. Also, it should be noted that the dynamic results are acquired in the diauxic and stationary phases of budding yeasts, which is essential for cytoophidium imaging but may affect the mobility of cytoophidia.

From the MSD analysis, we could also obtain the distribution of the diffusion coefficients *D* for the trajectories of the filaments. For the Glt1p filaments (*n* = 700), we found that most have a very low diffusion coefficient (<0.0025 μm^2^/s) (Fig. 5G), although some filaments with low intensities have relatively large coefficients (Fig. 5G, inset). This observation is reasonable because the filaments with low intensities had smaller Stoke radii and thus diffused faster in the viscoelastic cytoplasm crowded with macromolecules, which is illustrated by the fast diffusion of Fig. 5B (in contrast to Fig. 5C). For all six types of filament, we found that their diffusion coefficients ranged from 0.001 to 0.005 μm^2^/s (Fig. 5H) and are comparable with those reported by Zhou et al. (2011).

## Discussion

### Regulation of metabolism *via* filamentation

Filamentation appears to regulate CTPS enzymatic activity as suggested in previous studies (Aughey et al., 2014a, Aughey et al., 2014b, Barry et al., 2014, Noree et al., 2014). Thus, forming cytoophidia might serve as a complementary regulatory strategy for CTPS other than allosteric regulators or phosphorylation (Aughey et al., 2014a, Aughey et al., 2014b).

More metabolic enzymes have been verified to form filaments by our screening. Thus, further study of the regulation of these filaments may answer questions such as whether filamentation is a common way of enzyme regulation, and whether filamentation is an indicator of a different form of cell metabolism.

Take asparagine synthetase for example. It has already been documented that under amino acid deprivation or endoplasmic reticulum stress, asparagine synthetase expression is upregulated *via* putative ATF4 transcription regulation (Chen et al., 2004). Expression level change may result in a change to filament formation. Meanwhile, the other way around, it is worth identifying whether filament formation can regulate asparagine synthetase activity under amino acid deprivation. Filament formation may either be an indicator or an active regulator of a different metabolism situation. Since the upregulation of asparagine synthetase resulted in drug resistance in acute lymphoblastic leukaemia during chemotherapy (Aslanian et al., 2001), and a high asparagine synthetase expression level was detected in prostate cancer and was also related to aggressiveness of gliomas (Sircar et al., 2012, Panosyan et al., 2014), the detailed study of the regulation of asparagine synthetase may have important clinic implications.

### Growth conditions of budding yeast

Eukaryotic cell cycle is controlled in concert by specific growth factors and essential nutrients. In brief, *S. cerevisiae* utilises carbon sources (e.g., glucose or galactose) *via* fermentation and non-fermentable carbon sources (e.g., glycerol or ethanol) *via* respiration, respectively (Stahl et al., 2004). In the exponentially fermentative growth phase, ethanol produced is consumed during the second respiratory growth phase, post glucose exhaustion which is termed the diauxic shift. Once the ethanol is fully consumed, the yeast cells cease growing and shift into the stationary phase (Herman, 2002). Understanding of the growth phase transitions is vital as many studies of stationary phase are in fact conducted with cultures that are actually in the post-diauxic phase and thus, the conclusions made from these data might be misleading (Herman, 2002).

In our studies, we defined different growth phases of budding yeast based on previously documented studies (Allen et al., 2006, Davidson et al., 2011). In fact, there are two distinct fractions of cells, i.e., non-quiescence (NQ) and quiescence (Q) cells, in the stationary phase (Allen et al., 2006). Q cells can be characterised as dense, unbudded daughter cells; they are highly refractive by phase-contrast microscopy, thermotolerant, uniform in size and synchronously enter the mitotic cell cycle as compared to the NQ cells (Allen et al., 2006).

To decipher the regulation of cytoophidia in different growth phases, we analysed metabolic enzymes that involved in the biosynthetic pathways of amino acids (Asn1p, Asn2p and Glt1p) and pyrimidine (Ura7p and Ura8p). In common, these proteins shared a common domain that is composed of glutamine amidotransferase (GAT). GATs channel ammonia from a glutamine substrate to an acceptor substrate at the synthase site to produce an array of different aminated products (Mouilleron and Golinelli-Pimpaneau, 2007). GATs are grouped into distinct classes. Triad GATs (Class I) utilise histidine and glutamate residues to activate the cysteine thiol group. N-terminal nucleophile (NTN) (Class II) GATs contain the catalytic cysteine at the N-terminus and the α-amino group is required for the activation of thiol group. The open α/β structure fold in the glutaminase domain is common in most triad GATs, whereas, anti-parallel β-sheets are the main constituent in the NTN GAT counterpart. In budding yeast, CTPS (Ura7p and Ura8p) are categorised as Class I GATs, whereas asparagine synthetases (Asn1p and Asn2p) and glutamate synthase (Glt1p) are regarded as NTN GATs. Our data indicate that proteins grouped into different GAT classes can share the common ability to coalesce into cytoophidia.

The abilities of cells to detect detrimental changes in the environment and generate specific responses by reprogramming physiological and metabolic processes are pivotal for cell survival. Budding yeast has served as a good experimental model to investigate this biological feature under nutrient starvation (Broach, 2012). Is assembly of metabolic enzyme into cytoophidium a strategy for cell survival? As suggested in the previous study that cytoophidia serve as the storage depot of metabolic enzymes under nutrient stress (Petrovska et al., 2014), the changes in the abundance of yeast cells with glutamine-utilising cytoophidia and the length of this subcellular structure in these distinct growth phases remain as a major question. In this study, an increasing pattern of the abundance and length of cytoophidia were observed as the growth phase shifted from exponential to stationary phases. These observations illustrate that cytoophidium assembly serves as a mechanism to store metabolic enzymes when the cellular conditions are not favourable for growth. However, specific pathways that regulate the formation of cytoophidia remain unknown. Target of rapamycin (TOR) protein is a serine-threonine kinase that integrates various signals from intracellular and extracellular for the regulations of metabolism, growth and even survival of cell (Laplante and Sabatini, 2009). Speculatively, this central integrator of the availabilities of nutrients and growth factors might involve in the regulation of cytoophidium formation. As cytoophidia can be formed by enzymes regulating diverse metabolic processes and displayed heterogeneities, we proposed that the reorganizations of metabolic enzymes into cytoophidia are regulated by multiple signaling pathways.

### Implication of cytoophidia in human diseases

Within cells, metabolism is a highly coordinated and regulated process, which involves cooperation of many enzymes to accomplish critical functions. Metabolic enzymes are regulated at multiple levels. Abnormal metabolism contributes to several human diseases, including cancer, diabetes and obesity. Recently, we and other groups have demonstrated that the compartmentation *via* filamentation of the metabolic enzyme CTPS provides a novel mechanism for regulation of metabolic processes. We have shown that cytoophidium formation is regulated by its protein levels, glutamine availability, developmental cues and nutritional stress. Nevertheless, the finding of additional filament-forming proteins in this study highlights the complexity of intracellular compartmentation.

### Nuclear cytoophidia

It is unknown if Asn1p and Asn2p form the same structures, although their patterns in the cytoplasm and nucleus are very similar to each other. Given that both Ura7p/Ura8p and Asn1p/Asn2p are glutamine-utilising enzymes, it would be interesting to see whether the Asn1p/Asn2p filaments are similar to the Ura7p/Ura8p filaments, which are sensitive to glutamine deprivation or glutamine analogue treatment. Key questions include whether and how the Asn1p/Asn2p cytoophidia interact with the Ura7p/Ura8p cytoophidia, and whether and how all of these cytoophidia regulate glutamine metabolism *via* a common mechanism.

In summary, we perform a genome-wide screening of a GFP-tagged ORF collection in budding yeast and identify novel filament-forming proteins. We also demonstrate that filamentation of metabolic enzymes is very sensitive to growth condition. Since our large-scale screening is limited in a specific growth condition, this raises the possibility that many more filament-forming proteins might escape our detection. Moreover, we demonstrate that a few glutamine-utilising enzymes form cytoophidia both in the cytoplasm and the nucleus. Therefore, the identification of the cytoophidium and its kind provides a good opportunity to link metabolic pathways and subcellular organisation.

## Materials and methods

### Yeast strains and media

*S. cerevisiae* strains used in this study were derived from the yeast GFP clone collection comprising 4159 GFP-tagged ORFs (Huh et al., 2003). Each strain originates from a parent strain with the genotype *MATα his3Δ1 leuΔ0 met15Δ0 ura3Δ0* (*S288c*). All yeast strains were replicated into 96-well plates consisting of 150 μL of YPD media using a pin tool and sealed with MicroAmp™ adhesive film (Applied Biosystems, Foster City, CA, USA) to reduce well-to-well contaminations. All strains were grown at 32°C for 24 h for the screening of filament-forming proteins.

### Screening of filament-forming proteins

Cells were fixed in 4% paraformaldehyde for 10 min. The fixed cells were washed by PBS. Then cells were visualised under 40× objective on an inverted microscope (Nikon Eclipse Ti, Japan). Cells showing filaments were picked out for confocal imaging. For nucleus visualisation, Hoechst 33342 was added. Imaging of fixed samples was acquired under 63× objective on a laser-scanning confocal microscope (Leica TCS SP5 II confocal microscope, Germany).

### Quantification of filament-forming proteins

All yeast strains were grown overnight and diluted to an OD_600_ of ∼0.05 in 3 mL of YPD at 32°C in the incubator (300 r/min) and kept for 6, 24 and 168 h which reflected the exponential phase, diauxic phase and stationary phase, respectively. Cells were transferred into 1 mL microcentrifuge tubes and spun down for 1 min at 4000 r/min. The media were decanted and 100 μL of 4% paraformaldehyde were added to each tube to fix the cells for 10 min, and then the cells were washed once with PBS. A few microlitres of cell suspension were transferred onto the slides, followed by agarose gel to avoid movement of the cells during live imaging. The samples were covered with coverslips and inverted, and some pressure was applied to remove excess liquid. The coverslips were further sealed with nail polish to avoid the samples from drying. Once the nail polish had dried, the samples were ready for microscopic analysis.

The abundance (%) and averaged length (μm) of filaments-in cells in all three growth phases were compared and quantified by capturing images in at least four different areas containing a minimum of 100 cells each, using a laser-scanning Leica TCS SP5 II confocal microscope with a 63× oil objective lens. All images were taken at multiple focal planes and each of them was deconvolved and compressed into a single image. Image processing was carried out using ImageJ software. The experiments were repeated three times independently and data were presented as mean ± SD in the bar graph. The abundance and length of cytoophidia for all metabolic enzymes were analysed by One-Way Anova (multiple comparisons), except for Asn1p and Asn2p, which were analysed by Student's *t*-test (two-tailed).

### Protein-protein interaction analysis

Protein-protein interaction analysis was carried out by using the Search Tool for the Retrieval of Interacting Genes/Proteins (STRING) database version 10 (http://string-db.org/) (Szklarczyk et al., 2015). In the networks from STRING, nodes are proteins and edges are the predicted functional associations between proteins based on primary databases containing KEGG and GO, and primary literature. Various active prediction methods, such as neighbourhood, gene fusion, co-occurrence, coexpression, experiments, databases, homology and text mining, can be chosen for STRING analysis. In the current study, all of the prediction methods were used with medium confidence score 0.400.

### Live imaging

For time-lapse fluorescence imaging, cells were pipetted onto Glass Bottom Microwell Dishes (MatTek Corporation, Ashland, MA, USA) at stationary stage with appropriate dilution in PBS for the cells to be a single layer on the bottom. After incubating the cells at room temperature for 30 min, time-lapse videos were taken at 1.29 s intervals between each frame over 10 min of real time. Three videos for each sample have been taken with random fields containing filaments.

### Particle tracking and dynamic analysis

Tracking of the cytoophidia was performed by using the ImageJ plugin Particle Tracker (Sbalzarini and Koumoutsakos, 2005). In each frame, the cytoophidia were localized by adjusting the parameter radius and percentile in Particle Tracker software. A radius of 5–7 pixels and a percentile of 0.3%–0.5% were selected to capture the greatest number of cytoophidia that were clearly visible. The parameters of linking range and displacement were set to 2 frames and 5 pixels, respectively, to link the detected particles between frames. All the linking parameters for each kind of cytoophidia were checked and optimized manually.

For further dynamic analysis, only the trajectories longer than 15 frames (approximately 20 s) were selected. The MSD was calculated by the equation $\text{MSD}\left(\tau \right)=\langle {\left|r\left(t+\tau \right)-r\left(t\right)\right|}^{2}\rangle$, where *τ* is the time lag. Then the first 15 points of MSD were fitted by the power law $\text{MSD}\left(\tau \right)=\text{A}\cdot \tau^{\alpha }$. The exponent *α* indicates the nonlinear relationship of MSD with time, carrying information about the motion modes (Saxton and Jacobson, 1997): *α* ≈ 1 corresponds to free Brownian motion (i.e., free diffusion), *α* < 1 sub-diffusion (i.e., diffusion within a crowded medium), *α* > 1 super-diffusion (i.e., diffusion overlaid with deterministic motion), and *α* ≈ 2 directed motion. Similarly, the diffusion coefficient *D* is determined by fitting the 3 initial points of the MSD curves with MSD(τ)=4D·τ+c. All the analyses were performed using a user-defined program in Matlab.

## Figures and Tables

**Table 1:** Filament-forming proteins in *S. cerevisiae*

Protein	Name description	Biological process
**Acc1p**	**Acetyl-CoA carboxylase**	**Long-chain fatty acid biosynthetic process**
**Asn1p**	**Asparagine requiring**	**Asparagine biosynthetic process**
**Asn2p**	**Asparagine requiring**	**Asparagine biosynthetic process**
Cdc10p	Cell division cycle	Septin ring assembly
Cdc11p	Cell division cycle	Septin ring assembly
Cdc12p	Cell division cycle	Septin ring assembly
**Gcd1p**	**General control derepressed**	**Regulation of translational initiation**
Gcd2p	General control derepressed	Regulation of translational initiation
Gcd6p	General control derepressed	Regulation of translational initiation
Gcd7p	General control derepressed	Regulation of translational initiation
Gcn3p	General control nonderepressible	Regulation of translational initiation
**Gdb1p**	**Glycogen debranching enzyme**	**Glycogen catabolic process**
**Gdh2p**	**Glutamate dehydrogenase**	**Nitrogen compound metabolic process**
Gln1p	Glutamine metabolism	Glutamine biosynthetic process
Glt1p	Glutamate synthase	Glutamate biosynthetic process
**Pfk1p**	**Phosphofructokinase**	**Glycolytic process**
**Pfk2p**	**Phosphofructokinase**	**Glycolytic process**
Psa1p	GDP-mannose pyrophosphorylase	GDP-mannose biosynthetic process
Shs1p	Seventh homolog of septin	Septin ring assembly
Sui2p	Suppressor of initiator codon	Regulation of translational initiation
**Tsa1p**	**Thiol-specific antioxidant**	**Cell redox homeostasis**
Ura7p	Uracil requiring	CTP biosynthetic process
Ura8p	Uracil requiring	CTP biosynthetic process

The novel proteins identified in this study are in bold.

**Table 2:** KEGG pathways analysis of filament-forming proteins in STRING 10

KEGG pathway ID	Term	Number of genes	Gene name	*P*-value	*P*-value_FDR
sce250	Alanine, aspartate and glutamate metabolism	5	*ASN1, GLN1, ASN2, GDH2, GLT1*	4.06E-08	4.34E-06
sce3013	RNA transport	6	*GCD2, GCD1, GCN3, GCD7, GCD6, SUI2*	2.07E-07	1.11E-05
sce1100	Metabolic pathways	12	*ASN2, ACC1, ASN1, GLN1, PSA1, GLT1, GDH2, PFK2, URA7, GDB1, PFK1, URA8*	5.19E-07	1.85E-05
sce910	Nitrogen metabolism	3	*GLN1, GDH2, GLT1*	7.05E-07	1.89E-05
sce1120	Microbial metabolism in diverse environments	6	*ASN1, ASN2, GLN1, PFK1, PFK2, GLT1*	1.23E-05	2.62E-04
sce51	Fructose and mannose metabolism	3	*PFK1, PFK2, PSA1*	6.85E-05	1.22E-03
sce1110	Biosynthesis of secondary metabolites	6	*ASN2, PFK2, ASN1, PSA1, PFK1, GLT1*	2.12E-04	3.25E-03
sce1230	Biosynthesis of amino acids	4	*GLN1, PFK1, PFK2, GLT1*	6.90E-04	9.23E-03
sce52	Galactose metabolism	2	*PFK1, PFK2*	2.50E-03	2.98E-02
sce30	Pentose phosphate pathway	2	*PFK1, PFK2*	4.05E-03	3.94E-02
sce680	Methane metabolism	2	*PFK1, PFK2*	4.05E-03	3.94E-02
sce330	Arginine and proline metabolism	2	*GLN1, GDH2*	4.95E-03	4.41E-02

## Supplementary data

Fig. S1. Confirmation of filament-forming proteins in *Saccharomyces cerevisiae*.
