# Supplementary material for: Filamentation of Metabolic Enzymes in Saccharomyces cerevisiae
Source: J Genet Genomics. 2016 Jun 20;43(6):393–404. doi: 10.1016/j.jgg.2016.03.008 (PMC4920916; doi:10.1016/j.jgg.2016.03.008)
Supplement: Supplementary file 1 [file mmc1.docx]

**SUPPLEMENTARY DATA**

**Fig. S1**

**
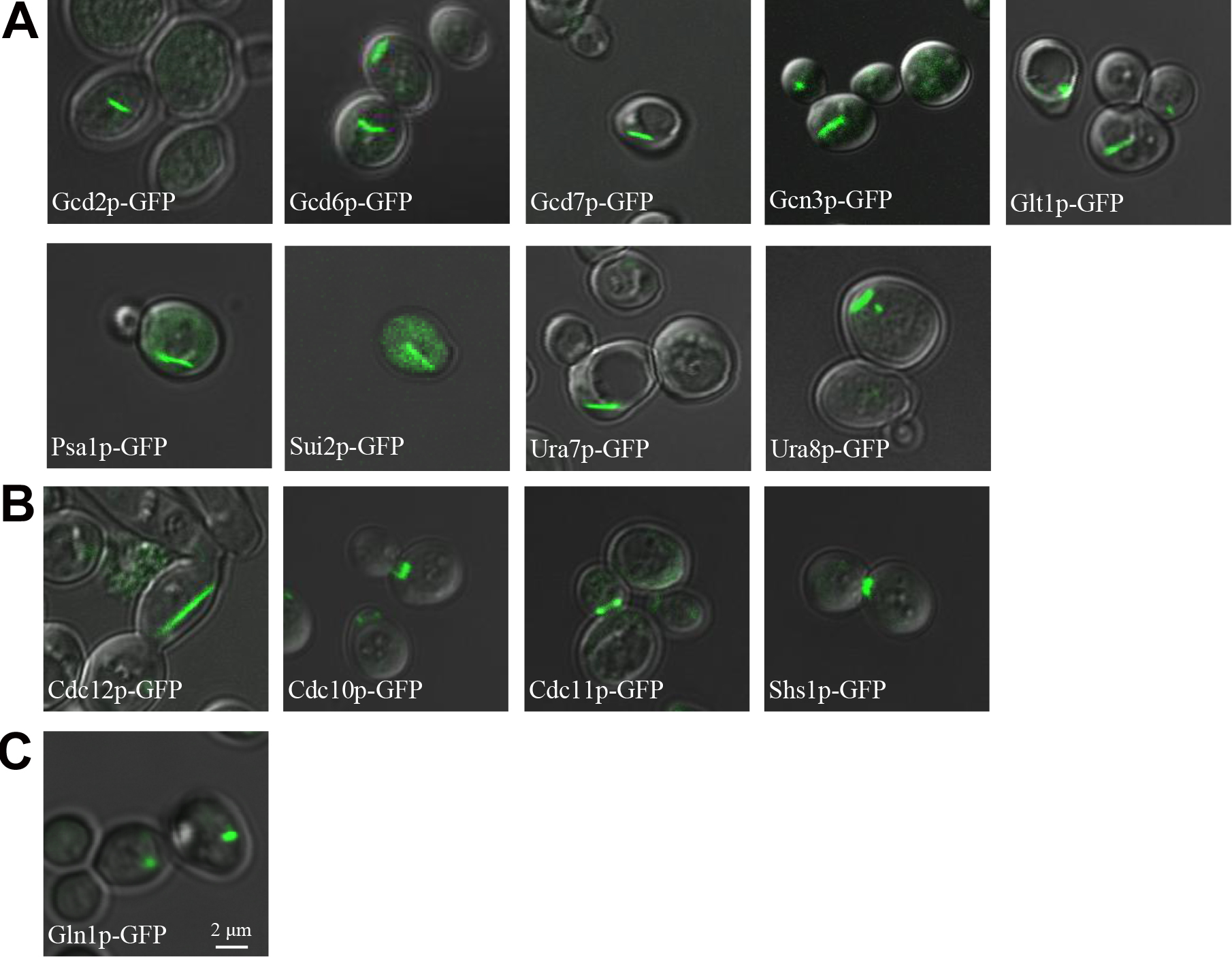
**

**Fig. S1. Confirmation of filament-forming proteins in *Saccharomyces cerevisiae*.**

**A：** Nine filament-forming proteins (Gcd2p, Gcd6p, Gcd7p, Gcn3p, Glt1p, Psa1p, Sui2p, Ura7p, and Ura8p) reported in a previous study ([Noree et al., 2010](#_ENREF_1)). **B：** Four filament-forming proteins (Cdc12p, Cdc10p, Cdc11p, and Shs1p) in septin complex. **C:** Gln1p (glutamine synthetase) can form short filaments and foci in *S. cerevisiae*, supporting a finding reported previously ([Petrovska et al., 2014](#_ENREF_2)). Scale bar, 2 µm.

**REFERENCES**

Noree, C., Sato, B.K., Broyer, R.M., Wilhelm, J.E., 2010. Identification of novel filament-forming proteins in Saccharomyces cerevisiae and *Drosophila melanogaster*. J. Cell Biol. 190, 541-551.

Petrovska, I., Nuske, E., Munder, M.C., Kulasegaran, G., Malinovska, L., Kroschwald, S., Richter, D., Fahmy, K., Gibson, K., Verbavatz, J.M., Alberti, S., 2014. Filament formation by metabolic enzymes is a specific adaptation to an advanced state of cellular starvation. eLife 3, e02409.
